# KGSD-Net: a knowledge graph syndrome differentiation network for syndrome classification

**DOI:** 10.3389/fmed.2025.1555781

**Published:** 2025-05-21

**Authors:** Guokai Zhang, Haoyu Jiang, Le Kuai, Bin Li, Chenxi Huang, Xiaoya Fei, Zhiyuan Huang

**Affiliations:** ^1^School of Optical-Electrical and Computer Engineering, University of Shanghai for Science and Technology, Shanghai, China; ^2^Department of Dermatology, Yueyang Hospital of Integrated Traditional Chinese and Western Medicine, Shanghai University of Traditional Chinese Medicine, Shanghai, China; ^3^Institute of Dermatology, Yueyang Hospital of Integrated Traditional Chinese and Western Medicine, Shanghai University of Traditional Chinese Medicine, Shanghai, China; ^4^Shanghai Skin Disease Hospital, Institute of Dermatology, School of Medicine, Tongji University, Shanghai, China; ^5^School of Informatics, Xiamen University, Xiamen, China; ^6^Xiamen Xianyue Hospital, Xianyue Hospital Affiliated with Xiamen Medical College, Fujian Psychiatric Center, Fujian Clinical Research Center for Mental Disorders, Xiamen, China

**Keywords:** electronic medical records, large-scale language model, knowledge graphs, traditional Chinese medicine, syndrome differentiation

## Abstract

The integration of electronic medical records (EMRs) in modern healthcare holds significant promise; however, traditional approaches to syndrome differentiation in Traditional Chinese Medicine (TCM) often encounter limitations due to incomplete data and inconsistent frameworks. This paper addresses these challenges by introducing a novel methodology that employs large-scale language models (LLMs) to extract relevant entities from an semi-structured TCM knowledge base, facilitating the construction of a dynamic TCM knowledge graph. By applying the DeepWalk method for latent knowledge graph embedding, hidden patterns essential for accurate diagnosis are uncovered. Furthermore, a combined entity linking approach is implemented to align this knowledge graph with diagnostic data extracted from EMRs, enhancing clinicians' insights through essential knowledge-based embeddings tailored specifically for syndrome differentiation tasks. Additionally, the integration of the BERT model with knowledge graph embedding technologies strengthens dialectical reasoning within TCM practice and demonstrates superior performance on specialized datasets compared to prior methodologies.

## 1 Introduction

Modern medicine has advanced electronic medical records (EMRs) by effectively integrating online and offline management systems, improving efficiency and accessibility compared to traditional record-keeping methods. As EMR systems continue to evolve within contemporary healthcare, traditional Chinese medicine (TCM) — a vital component of global medicine is also gradually being standardized. TCM electronic medical records (TCM-EMRs) typically encompass essential diagnostic information, including physical examinations, chief complaints, syndrome identification, treatment plans, and herbal prescriptions (1–3). This comprehensive data forms the foundation for TCM practitioners to perform accurate syndrome differentiation and develop effective treatment strategies.

However, it appears that there are still gaps between the technological potential of EMRs and their practical application in the specific context of TCM. The complexities involved in accurately capturing patient symptoms and their corresponding syndromes suggest a need for more sophisticated analytical approaches. Currently, many methodologies tend to rely on basic statistical analyses or rule-based systems, which may not fully encompass the multifaceted nature of TCM diagnostics. To bridge this gap, integrating artificial intelligence techniques into the TCM framework effectively enhances syndrome differentiation accuracy and efficiency. This is achieved by leveraging machine learning and deep learning models to extract valuable insights from TCM-EMRs while also automating routine data entry and processing tasks. Furthermore, incorporating natural language processing (NLP) capabilities improves the extraction of key clinical features from unstructured diagnostic narratives in patient records. Besides, the shift toward evidence-based practice supported by intelligent systems not only aligns with broader trends in contemporary healthcare but also honors the holistic principles inherent in TCM practices. By combining standardized clinical terminologies with advanced computational techniques, this approach promotes consistency in diagnosis and enables practitioners to tailor treatments more precisely according to individual patient circumstances.

Despite advancements in TCM, challenges in syndrome differentiation persist due to reliance on manual data entry and basic processing, leading to incomplete information that hinders clinicians' decisions, especially in complex cases. Additionally, the lack of standardized frameworks for integrating diverse data sources contributes to inconsistencies in diagnosis and treatment. To address these challenges, in this paper, an innovative approach called Knowledge graph syndrome differentiation network (KGSD-Net) is proposed that utilizes large-scale language models (LLMs) to extract critical information from semi-structured TCM knowledge bases. This process facilitates the construction of a dynamic TCM knowledge graph, serving as a centralized repository that enhances the visualization of connections between symptoms, diagnoses, and treatments. Furthermore, employing the DeepWalk method for latent knowledge graph embedding reveals essential patterns fundamental to accurate diagnosis. A unified entity linking strategy further aligns this knowledge graph with diagnostic data extracted from EMRs, thus creating a seamless integration of structured clinical data with traditional practices. Such advancements provide knowledge-based latent embeddings tailored specifically for syndrome differentiation tasks, offering clinicians deeper insights into their decision-making processes. Moreover, by integrating BERT models with knowledge graph embedding technologies, the sophistication of dialectical tasks within TCM practice is efficiently enhanced. Validation on specialized TCM datasets demonstrates that this comprehensive method outperforms previous approaches, particularly in terms of performance in syndrome differentiation. Overall, the main contributions of this paper could be summarized as follow:

– A dynamic TCM knowledge graph is constructed using LLMs, enabling the extraction of effective entities from an semi-structured TCM knowledge base to create a comprehensive repository of vital information.
– The DeepWalk method for latent knowledge graph embedding is employed to uncover hidden patterns essential for accurate syndrome differentiation, providing deeper insights into the relationships among symptoms, diagnoses, and treatments.
– A combined entity linking approach is utilized to align the knowledge graph with diagnostic data extracted from EMRs, facilitating seamless integration of structured clinical data with traditional TCM practices.
– Validation on specialized TCM datasets confirms that the proposed approach demonstrates superior performance in syndrome differentiation compared to previous methods, showcasing its effectiveness in enhancing clinicians' decision-making processes.

## 2 Related works

NLP has gained significant attention in the medical field for enhancing patient management and facilitating knowledge discovery. Its applications, including information extraction, text classification, and clinical decision support, are transforming healthcare practices. For instance, speech recognition technology is effectively used to transcribe prescriptions swiftly into Electronic Health Records (EHR) (4). Additionally, paper-based medical records have been converted into searchable and manageable EMRs (5). This transition not only streamlines diagnoses but also supports research efforts for healthcare professionals by making relevant data more accessible and easier to analyze. As a branch of artificial intelligence, machine learning employs various algorithms to uncover potential and meaningful patterns from large datasets. For example, Tang et al. (6) conducted a comprehensive analysis using frequency analysis, association rules, and hierarchical clustering to explore the four diagnostic methods. Similarly, Lu et al. (7) devised a computer-aided system utilizing machine learning techniques to assess the severity of sublingual varicosity. Additionally, to reduce the subjectivity inherent in electronic medical records, Fan et al. (8) used a random forest algorithm to extract and select various features from 466 tongue images, successfully classifying patients based on two different TCM symptoms related to gastric conditions.

At the same time, deep learning methods automate semantic extraction through various neural network architectures. For instance, Teng et al. (9) developed a Symptom-State Graph Convolutional Network (SSGCN) that integrates symptoms and state elements, effectively embedding the inherent logic of TCM diagnosis into a prescription graph. They then trained a multi-layer perceptron (MLP) to classify different syndromes. In addition, Chen et al. (10) created a TCM-BERT-CNN model that combines BERT and CNN for end-to-end TCM evidence identification, transforming symptom input into classifications of evidence output. Nevertheless, models like RoBERTa (11), ERNIE (12), and XLNET (13) often require additional pre-training for downstream tasks, which can significantly increase both time and cost. Furthermore, recent studies suggest that augmenting models with relevant knowledge beyond the input data can greatly improve performance across a range of applications, including disease diagnosis (14), document classification (15), sentiment analysis (16), and even question-answering systems (17). In this context, Castellano et al. (18) proposed an innovative classification method that uses knowledge graphs in conjunction with deep learning techniques. Similarly, Yang et al. (19) proposed a syndrome differentiation decision-making system (DSDS) based on a TCM knowledge graph. By decomposing medical records into multiple symptom nodes and performing link prediction within the knowledge graph, the system enables automatic inference of syndromes, demonstrating the effectiveness of knowledge graphs in intelligent TCM diagnosis.

## 3 Methodology

**Figure 1:**
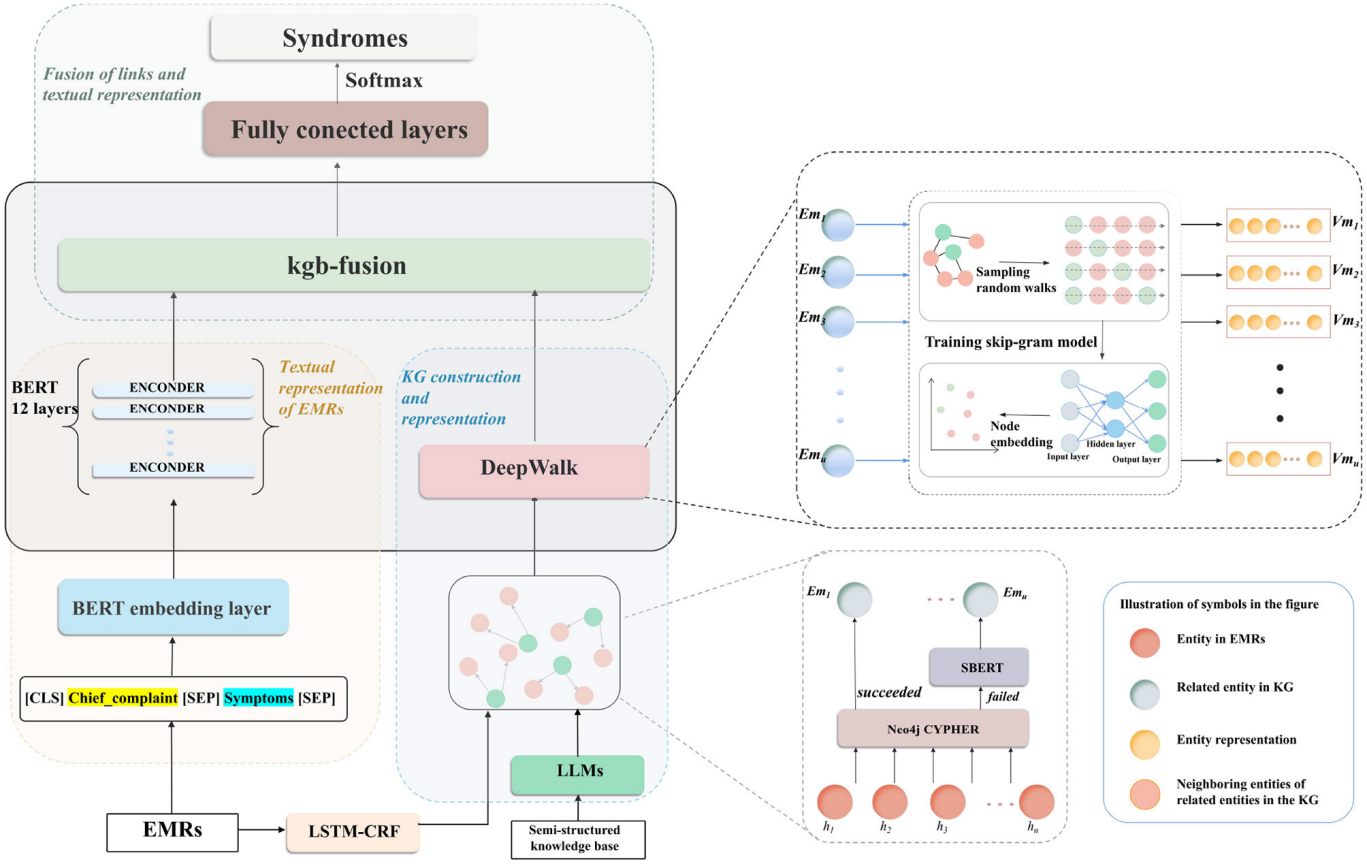
Overview of the KGSD-Net. It consists of KG construction and representation, textual representation of EMRs, and kgb-fusion. LSTM-CRF processes structured EMRs, while LLMs handle semi-structured data to build the KG. BERT extracts textual features from EMRs. KG links and textual representations are fused using kgb-fusion for syndrome prediction.

As shown in Figure 1, the proposed KGSD-Net consists of three main processes: KG construction and representation, textual representation of EMRs, and fusion of links with textual representation. In the first process, KG construction and representation utilizes LSTM-CRF (20) to handle structured EMRs while concurrently processing the semi-structured knowledge base through LLMs to construct the KG. Next, during the textual representation of EMRs, the BERT model is employed to extract textual representations of chief complaints and symptom information. Finally, we fuse the KG links with the textual representations by adopting knowledge graph embedding fusion (kgb-fusion) techniques and subsequently pass these integrated outputs to a fully connected layer with softmax activation for predicting syndromes.

### 3.1 KG construction and representation

The process begins with the integration of semi-structured and structured knowledge bases. First, we define the entities and relationships for the KG, and then we utilize LSTM-CRF and LLMs to enhance the understanding of symptoms in electronic medical records. Following this, KG fusion and cleaning are implemented during the integration of the KG to standardize the entities. This results in a compact KG that encompasses 148 syndromes and associated diagnostic information, which is subsequently stored in Neo4j. Finally, the DeepWalk model is employed for KG representation learning, effectively transforming implicit knowledge into vector representations.

The attributes of symptoms, syndromes, and herbs from SymMap provide the foundation for defining the properties of syndrome entities, as illustrated in Table 1. This initial step establishes essential attribute terms for core entities such as drugs, syndromes, and symptoms, clarifying their quantity and interrelationships. Additionally, supplementary knowledge bases offer valuable descriptions and definitions for secondary entity categories, including formulas and treatment methods. Consequently, a comprehensive KG is created, encompassing core entity categories along with their associated attributes—specifically symptoms, syndromes, formulas, treatment methods, herbs, and disease mechanisms while also incorporating secondary categories such as drug components and affected areas. Following this integration of entities, various forms of entity extraction components are developed to accommodate both semi-structured and structured knowledge bases.

**Table 1:** Entities with multiple attributes and their counts.

**Entity type**	**Entity attributes**	**Attribute numbers**
Syndrome	Syndrome_name, Syndrome_English, Syndrome_PinYin, Syndrome_definition	4
Symptoms	TCM_symptom_name, Symptom_PinYin, Symptom_definition, Symptom_locus, Symptom_property	5
Herb	Chinese_name, Pinyin_name, English_name, Properties_Chinese, Meridians_Chinese	5

#### 3.1.1 LSTM-CRF for structured knowledge base

To further enhance and expand the knowledge graph centered on the four diagnostic methods in TCM, we carry out entity recognition and supplementary annotation for diagnostic records based on the TCM-SD dataset, selecting and pre-processing data from five representative cases within each of the 85 syndrome categories to refine the entity recognition process. Afterwards, the data is used to train an LSTM-CRF model that effectively combines sequence learning and classification for entity recognition. In this context, the annotated input sequence is represented as *X* = (*x*_1_, *x*_2_, …, *x*_*n*_), where each *x*_*n*_ corresponds to a token in the input text. The objective of the LSTM-CRF model is to predict the optimal label sequence *Y* = (*y*_1_, *y*_2_, …, *y*_*n*_), with *y*_*n*_ being the label assigned to each corresponding token *x*_*n*_:


(1) $$\begin{array}{l} Y=f_{\text{LSTM-CRF}}\left(X\right)=\text{CRF}\left(\text{LSTM}\left(X\right)\right) \end{array}$$

In this process, the input sequence *X* is first processed through the LSTM layer, which captures sequential dependencies between tokens and allows the model to learn context-aware representations. Subsequently, the output from the LSTM is passed through the CRF layer, optimizing the label sequence *Y* by considering interdependencies between labels.

#### 3.1.2 LLMs for semi-structured knowledge base

OpenAI's API is utilized for automated entity extraction from the semi-structured TCM-SD knowledge. Initially, the dataset is prepared and formatted to comply with the API requirements. The input sequence, denoted as


(2) $$\begin{array}{l} \hat{X}=\left({\hat{x}}_{1},{\hat{x}}_{2},\ldots ,{\hat{x}}_{\hat{n}}\right) \end{array}$$

is structured to specify the target entity categories for extraction. Upon executing the API call, the extraction process yields the output sequences Ŷ, which consist of the identified entities, specifically the symptom entities *E*_*Sz*_ and syndrome entities *E*_*Sh*_. The process can be mathematically represented as follows:


(3) $$\hat{X}\overset{g}{\to }\hat{Y}\to (E_{Sz},R,E_{Sh})$$

where *g* denotes the extraction function that identifies and extracts the entities, and *R* represents the defined relationships between the extracted entities. Once the entities are extracted, they are systematically processed and organized into a structured representation *T*:


(4) $$\begin{array}{l} T=\left(E_{Sz},R,E_{Sh}\right) \end{array}$$

Through differentiated entity extraction methods applied to the original knowledge base, the final results include primary categories and their attributes, such as symptoms, syndromes, prescriptions, treatments, and disease mechanisms, as well as secondary entity categories like drug ingredients, affected locations, and diagnostic details from tongue and pulse examinations. Additionally, the relationships between entities reflect the intrinsic connections between different types of entities and form the foundation of the combined entity linking approach. Therefore, in line with the TCM diagnostic process, we have defined seven entity relationships based on observation and listening, as shown in the schema diagram of the knowledge graph in Table 2.

**Table 2:** Entity pairs and their relationship types.

**Entity pairs**	**Relationship types**
Syndrome-Disease	Has_disease
Syndrome-Symptoms	Has_symptom
Syndrome-Treatment	Has_treatment
Treatment-Drugs	Common pharmaceuticals
Drugs-Flavor	Has_flavor
Drugs-Form	Dosage form
Disease-Patients	Common patients
Disease-Dgroup	IspartofD
Drugs-Fgroup	IspartofP

#### 3.1.3 KG fusion and cleaning

**Figure 2:**
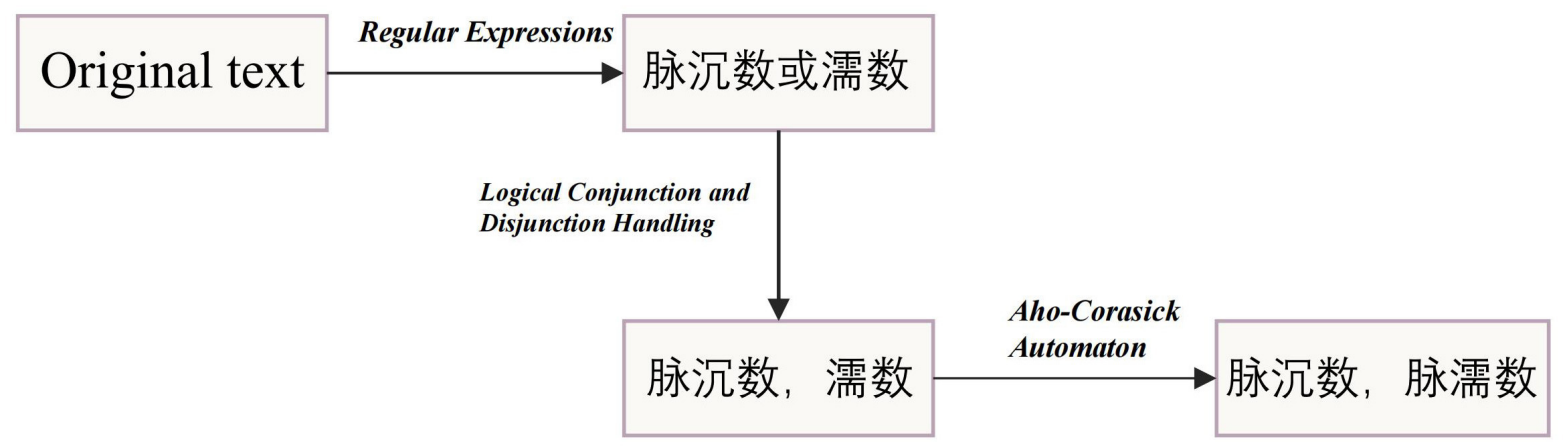
Example of symptom label processing process.

**Figure 3:**
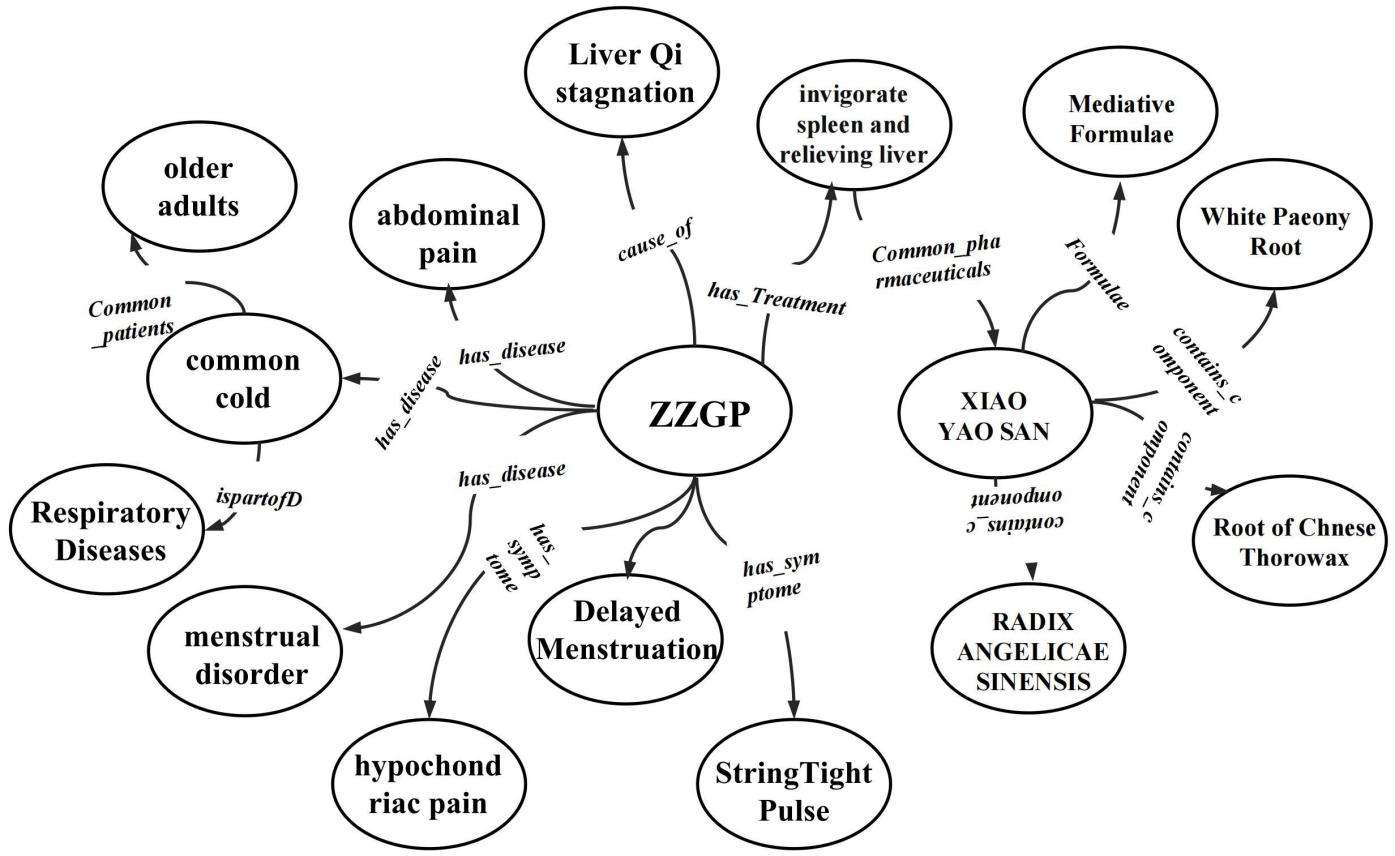
All type nodes and relationship types of TCMKG are listed, centering on the syndrome of Liver-qi stagnation and Spleen deficiency (ZZGP).

After completing entity extraction, let *KG*_1_, *KG*_2_, *KG*_3_ represent knowledge graphs from different sources, and the *KG* be denoted as *KG* = (*V, R*), where *V* is the set of nodes and *R* is the set of relationships. Each node *v*_*i*_ ∈ *V* is connected to other nodes via specific relationship types *r* ∈ *R*. The merged knowledge graph *KG* can be expressed as:


(5) $$\begin{array}{l} KG=f\left(KG_{1},KG_{2},KG_{3}\right) \end{array}$$

where *v*_*fused*_ = merge(*v*_1_ ∈ *KG*_1_, *v*_2_ ∈ *KG*_2_, *v*_3_ ∈ *KG*_3_) for nodes, and *r*_*fused*_ = merge(*r*_1_ ∈ *KG*_1_, *r*_2_ ∈ *KG*_2_, *r*_3_ ∈ *KG*_3_) for relationships. Notably, variations in entity descriptions and recognition can lead to confusion and redundancy in the knowledge base, affecting the accuracy of the knowledge graph due to diverse models and data sources (21). To address this, our model splits, reorganizes, deletes, and merges entities with overlapping meanings, particularly focusing on syndromes and symptoms. Specifically, the process, illustrated in Figure 2, begins by using regular expressions to locate symptom texts in the knowledge base. Logical Conjunction and Disjunction Handling is employed to deal with entities that have multiple meanings, and then entity names are matched to determine whether two or more entities represent the same knowledge concept. After that, a symptom vocabulary is sourced from relevant websites, a standard syndrome vocabulary is obtained from the *Classification and Codes of Diseases and Patterns of Traditional Chinese Medicine*, and an Aho-Corasick automaton (22) is used to normalize all symptoms and syndromes. Finally, these identical knowledge nodes are integrated into a single unique node, forming a multi-source knowledge base centered around syndromes, with the entire KG containing 5,063 nodes and 10,249 triples. Ultimately, we constructe a Traditional Chinese Medicine Knowledge Graph (TCMKG) centered on syndrome-type nodes, as shown in Figure 3, where we present all types of nodes and their relationships within TCMKG, with a focus on the syndrome of Liver-qi stagnation and Spleen deficiency.

#### 3.1.4 KG representation

To obtain knowledge graph embeddings that illustrate the connections between entity nodes, we utilize the DeepWalk algorithm for representation learning. This process involves two primary steps: first, extracting neighboring nodes to construct an adjacency list, and second, training the node sequences generated from random walks using the Skip-gram model. Specifically, the nodes and relationships extracted from the knowledge graph form a subgraph *G*′, and an adjacency list *A* is constructed to describe the graph structure:


(6) $$\begin{array}{l} A\left(v_{i}\right)=\left\{v_{j}|\left(v_{i},v_{j}\right)\in R\right\} \end{array}$$

This adjacency list serves as the basis for subsequent random walks. For each node *v*_*i*_, multiple random walks are initiated from the node, generating sequences that capture the graph's connectivity. The resulting node sequence *W*(*v*_*i*_) is expressed as:


(7) $$\begin{array}{l} W\left(v_{i}\right)=\left(v_{i_{1}},v_{i_{2}},\ldots ,v_{i_{T}}\right) \end{array}$$

where *T* represents the length of the walk. At each step, the next node *v*_*i*_*k*+1__ is randomly selected from the neighbors of the current node *v*_*i*_*k*__. These generated sequences serve as input for the Skip-gram model, which aims to maximize the probability of observing a context node *v*_*u*_ given a target node *v*_*w*_ within the walks. This objective is formalized by the following function:


(8) $$\begin{array}{l} L=\max\underset{w\in W}{\sum }\underset{u\in N\left(w\right)}{\sum }\log P\left(u|w\right) \end{array}$$

Here, *P*(*u*∣*w*) denotes the probability of observing context node *u* given target node *w*, defined by the softmax function:


(9) $$\begin{array}{l} P\left(u|w\right)=\frac{\exp\left({\text{V}}_{u}\cdot {\text{V}}_{w}\right)}{\sum_{v\in V}\exp\left({\text{V}}_{v}\cdot {\text{V}}_{w}\right)} \end{array}$$

In this equation, **V**_*u*_ and **V**_*w*_ are the embedding vectors for nodes *u* and *w*, respectively. By optimizing this objective function, the model learns node embeddings **V**_*m*_ and stores them in the attributes of each entity node for efficient querying and retrieval.

### 3.2 Textual representation of EMRs

The EMRs representation module consists of a BERT layer and an entity linking layer, where BERT extracts features from EMRs while SBERT model facilitates entity linking. To incorporate knowledge-embedded information, the input data undergoes modifications, beginning with entity recognition processing before it is fed into BERT. Additionally, an LSTM-CRF model trained on the manually labeled TCM-SD dataset identifies complaints, symptoms, and diseases. Consequently, inputs to the EMRs are formatted as [CLS] disease name, chief complaint [SEP] history [SEP], utilizing special symbols specific to BERT. Formally, given an input sequence **B** = [*b*_1_, *b*_2_, …, *b*_*s*_], BERT produces a sequence of embeddings **H** = [*h*_1_, *h*_2_, …, *h*_*s*_], where:


(10) $$\begin{array}{l} h_{s}={\text{TransformerLayer}}_{l}\left(b_{s},\text{B}\right) \end{array}$$

where TransformerLayer_*l*_ denotes the *l*-th transformer layer. For text classification tasks, the hidden state of the [CLS] token in the last layer is typically used as the representation of the entire sequence, as follows:


(11) $$\begin{array}{l} {\text{V}}_{\text{[emrs]}}={\text{H}}_{\text{[CLS]}}^{\left(L\right)} \end{array}$$

here, ${\text{H}}_{\text{[CLS]}}^{\left(L\right)}$ represents the hidden state of the [CLS] token in the *L*-th (final) layer.

### 3.3 Kgb-fusion of links and textual representation

**Figure 4:**
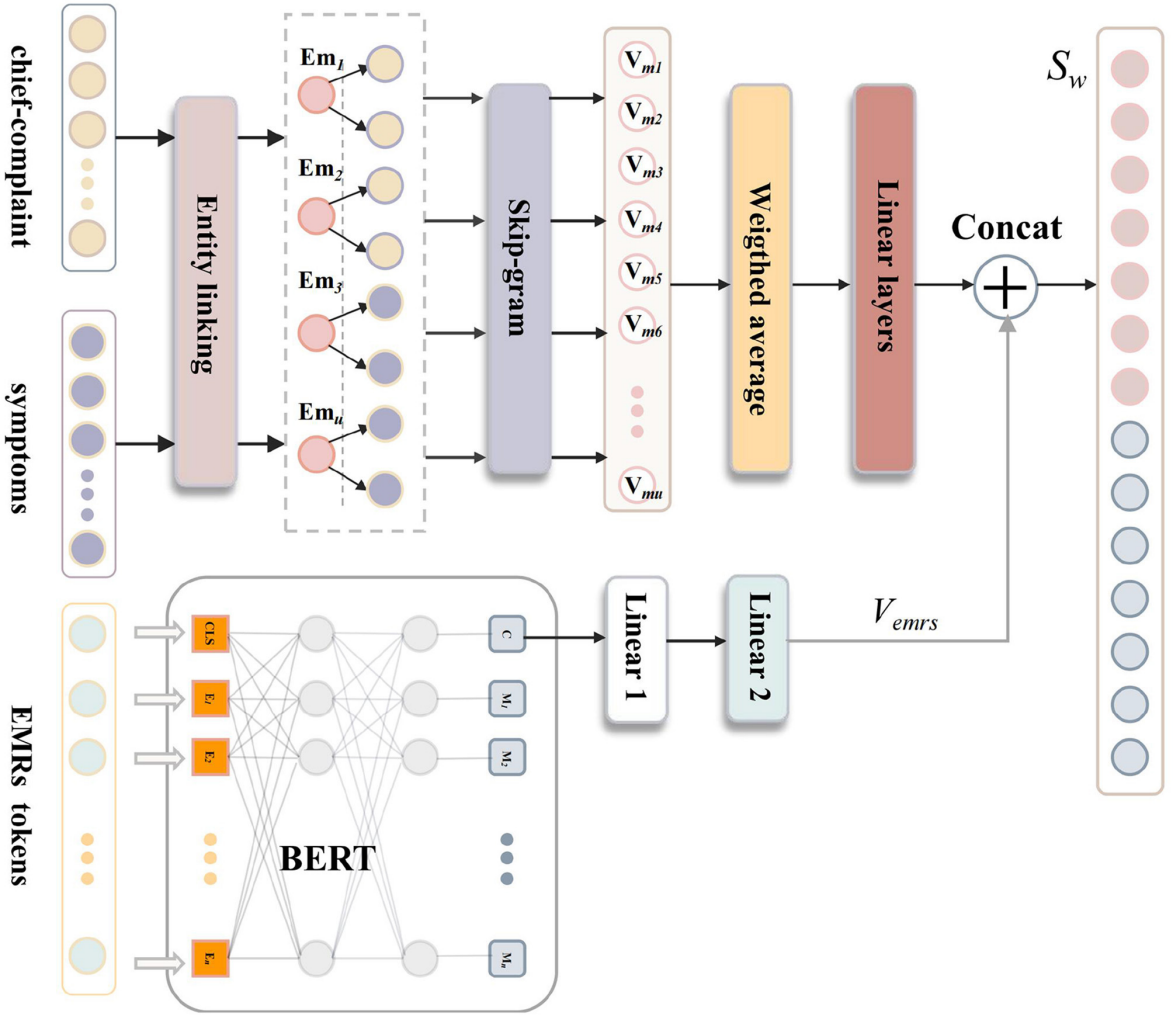
The kgb-fusion integrates knowledge graph data with BERT outputs to produce a fused vector for multi-class classification. This process involves entity linking through both hard matching and semantic similarity assessments. Ultimately, it results in a transformed knowledge embedding vector, which is then concatenated with the text representation to form the final input for classification.

The purpose of the kgb-fusion is to integrate information from the knowledge graph with outputs from BERT, creating a fused vector that is utilized for multi-class classification tasks. The specific process of kgb-fusion is illustrated in Figure 4. To enhance the accuracy of entity linking, a semantic model has been incorporated into the combined entity linking process. Initially, during the entity linking process, a “hard match” is performed using Neo4j's Cypher queries. If this match fails, the SBERT model (23) is employed to extract information from relevant nodes for computing semantic similarity. This process can be expressed as follows:


(12) $$Em=\{\begin{array}{ll} e_{\text{matched}}, & \text{if CypherMatch succeeds}\\ e_{\text{sbert}}, & \text{if CypherMatch fails} \end{array}$$

Here, *e*_matched_ represents the entity node that is successfully matched using Neo4j's Cypher query. *e*_sbert_ is the most likely node obtained by invoking SBERT when CypherMatch fails. Based on the selection criteria, the final linked entity *E*_*m*_ is determined. After identifying relevant nodes, their *V*_*m*_ representation vectors stored in node attributes are retrieved. Given that the knowledge graph primarily employs a “multi-symptom-multi-syndrome” triplet format, multiple data points could be obtained during this linking phase. Consequently, a weighted average is calculated based on the frequency of each relevant node's occurrence across all nodes to derive the final knowledge embedding vector. This final knowledge embedding vector is computed as follows:


(13) $$\begin{array}{l} {\text{V}}_{\text{kg}}=\frac{\sum_{i=1}^{u}f_{i}\cdot {\text{V}\text{m}}_{i}}{\sum_{i=1}^{u}f_{i}} \end{array}$$

Here, **V**_kg_ represents the final knowledge embedding vector, **Vm**_*i*_ denotes the embedding vector of the *i*-th relevant node, *f*_*i*_ indicates the frequency of the *i*-th relevant node, and *u* signifies the total number of relevant nodes. Following this step, the final representation vector obtained from the combined entity linking approach undergoes linear transformation before being concatenated with the text representation vector. Ultimately, the fused vector *S*_*w*_ undergoes another linear transformation to reduce its dimensionality from hidden layer size to match the number of labels:


(14) $$\begin{array}{l} S_{w}=\text{Concat}\left(\text{Linear}\left(V_{\text{kg}}\right),V_{\text{emrs}}\right) \end{array}$$

## 4 Result

### 4.1 Dataset

The dataset utilized in this paper is the proposed standardized dataset for TCM-SD identification. In each syndrome category, 5 records are extracted to supplement the knowledge graph and following this extraction, the composition of the dataset is updated to minimize the impact on minority classes. The revised dataset includes a total of 85 categories based on syndromes, containing 62,208 entries. Notably, the largest category contains 7,912 entries, while the smallest has only 25 entries, leading to an extremely unbalanced distribution.

### 4.2 Implementation details

Data cleaning and filtering are performed on the medical history terms in the dataset to eliminate duplicate entries, symbols, and timestamps that have minimal impact on identification. This preprocessing phase establishes a solid foundation for model development by ensuring high data quality. The training parameters for BERT include a hidden size of 768, a maximum position embedding of 512, and configurations of 15 epochs, 12 attention heads, and 12 hidden layers. Additionally, the maximum input length is set to 128, with a learning rate of 2 × 10^−5^ and a batch size of 16. Building on this setup, the experiments aim to determine an optimal knowledge embedding dimension of 128 while establishing a maximum DeepWalk step length of 20. All computational tasks are executed using a GEFORCE RTX 2070 GPU, providing robust support for these experiments. More details could be referred to Table 3.

**Table 3:** Model parameters and values.

**Parameter**	**Value**
Loss function	CrossEntropyLoss
Optimizer	AdamW
Batch size	16
Learning rate	2 × 10^−5^
Weight decay	0.02
Max seq length	128
KG embedding	384
Number of epochs	10
BERT model	Bert-base-chinese

Evaluation metrics for multiclass classification tasks include accuracy, macro-precision, macro-recall, and macro-F1. To define these metrics clearly, TP (True Positive) represents the total number of positive samples correctly predicted by the model, while TN (True Negative) refers to the total number of negative samples accurately identified. Additionally, FP (False Positive) indicates the number of negative samples incorrectly predicted as positive, and FN (False Negative) denotes the number of positive samples misclassified as negative.

#### 4.2.1 Accuracy

Accuracy (ACC) is defined as the ratio of correctly predicted samples to the total number of samples, as shown in Equation 15:


(15) $$\begin{array}{l} \text{ACC}=\frac{TP+TN}{TP+TN+FP+FN} \end{array}$$

#### 4.2.2 Macro-f1

The macro-f1 score is calculated by first averaging the precision and recall for all classes. Subsequently, the f1 score for each class is computed as the harmonic mean of its precision and recall. This process is summarized in Equation 16:


(16) $$\begin{array}{l} \text{Macro-f1}=\frac{1}{N}\overset{N}{\underset{k=1}{\sum }}\frac{2\cdot {\text{Precision}}_{k}\cdot {\text{Recall}}_{k}}{{\text{Precision}}_{k}+{\text{Recall}}_{k}} \end{array}$$

#### 4.2.3 Macro-precision

Macro-precision assesses the accuracy of a classifier's predictions across all classes by first calculating the precision for each individual class. This is then averaged to provide a comprehensive evaluation, as illustrated in Equation 17:


(17) $$\begin{array}{l} \text{Macro-precision}=\frac{1}{N}\overset{N}{\underset{k=1}{\sum }}\frac{TP_{k}}{TP_{k}+FP_{k}} \end{array}$$

Here, *N* represents the total number of classes, while *TP*_*k*_ and *FP*_*k*_ denote the true positives and false positives for class *k*, respectively.

#### 4.2.4 Macro-recall

Macro-recall evaluates a classifier's ability to accurately identify all relevant instances across different classes. To calculate it, the recall for each class is computed individually and then averaged, as demonstrated in Equation 18:


(18) $$\begin{array}{l} \text{Macro-recall}=\frac{1}{N}\overset{N}{\underset{k=1}{\sum }}\frac{TP_{k}}{TP_{k}+FN_{k}} \end{array}$$

### 4.3 Ablation study

An ablation study compares the classification performance of three models: the KG-only model, the BERT-only model, the KGSD-Net-noST model, and the proposed KGSD-Net. The KG-only model refers to the version of KTSD that has removed the BERT module for representing medical texts in EMRs. Meanwhile, the BERT-only model indicates the version where the knowledge graph representation and linking modules have been omitted. The KGSD-Net-noST model eliminates the SBERT model and relies exclusively on Neo4j lookups for entity linking. In this setup, if no entity is linked, the resulting knowledge embedding becomes a 384-dimensional zero vector. As shown in Table 4, the KG-only model underperforms relative to the BERT model on the TCM-SD dataset, indicating that relying solely on knowledge representation vectors offers limited advantages for syndrome differentiation tasks. This limitation arises from the high redundancy in TCM symptom records, where overlapping symptoms may correspond to different syndrome categories. In contrast, the combined approach leveraging both KG and BERT achieves superior classification results. This improvement is likely due to knowledge embeddings compensating for gaps in TCM-specific data during BERT's pretraining, while also addressing the shortcomings of knowledge-only methods in comprehensively interpreting diagnostic records. At the same time, the KGSD-Net-noST model still outperforms the BERT model, with Macro-f1 and Macro-precision improving by 3.36% and 3.65%, respectively. This finding demonstrates that the knowledge graph effectively contains entities consistent with the dataset. However, when compared to the complete KGSD-Net, the performance of KGSD-Net-noST shows a certain decline. These results suggest that disparities in descriptions within the knowledge graph remain significant, and employing SBERT model for semantic matching substantially enhances entity linking, thereby improving the effectiveness of the knowledge embeddings.

**Table 4:** Ablation experiment without different parts.

**Model**	**ACC**	**Macro-F1**	**Macro-precision**	**Macro-recall**
BERT-only	0.801	0.528	0.558	0.532
KG-only	0.571	0.282	0.276	0.289
KGSD-Net-noST	0.805	0.561	0.594	0.531
KGSD-Net	0.826	0.585	0.652	0.555

### 4.4 Performance on different KG-embedding dimension

The most suitable dimension for KG embeddings is explored under equivalent experimental conditions. Taking into account the computation time and performance of SBERT model in the linking component, 768 dimensions are set as the upper limit. The models are then compared with KG-embedding dimensions of 64, 128,384, 768 on the TCM-SD dataset. Experimental results, as shown in Table 5, indicate that the 384-dimensional KG embedding achieves the best classification performance. This could be attributed to the fact that this dimensionality matches the output vector size of SBERT model used in the linking module, resulting in more effective entity linkage. Furthermore, minimizing the dimensional disparity between text representation vectors and KG embeddings facilitates better information retention from KG embeddings during kgb-fusion.

**Table 5:** Performance comparison with different KG embedding dimensions.

**KG dimension**	**ACC**	**Macro-F1**	**Macro-precision**	**Macro-recall**
64	0.804	0.541	0.587	0.502
128	0.815	0.570	0.611	0.534
384	0.826	0.585	0.652	0.555
768	0.806	0.576	0.630	0.531

### 4.5 Performance on different fusion methods

**Figure 5:**
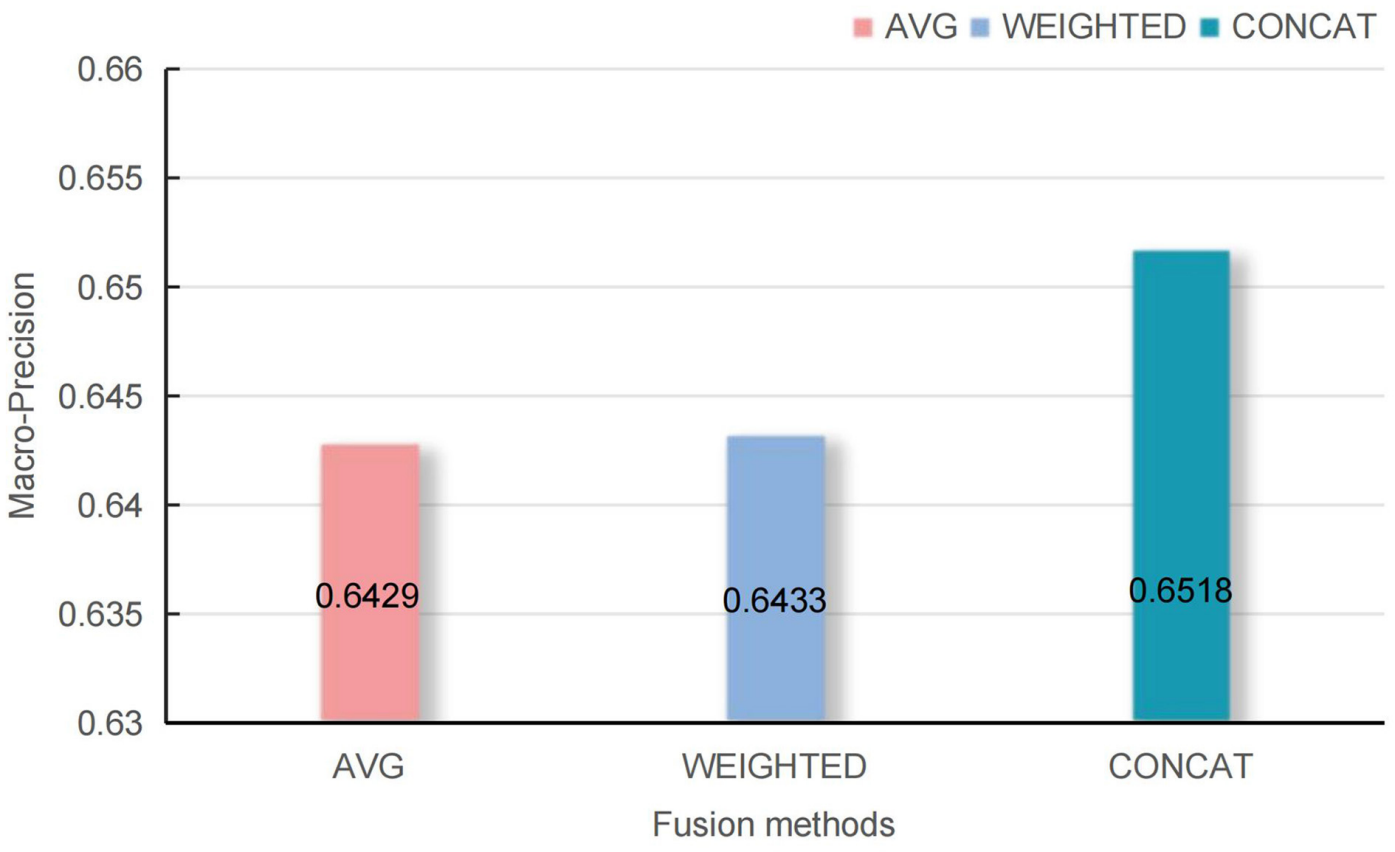
The figure illustrates the Macro-precision achieved by integrating *V*_emrs_ and KG representations *V*_kg_ using average (AVG), weighted sum (WEIGHTED), and concatenation (CONCAT) methods. Among these, the CONCAT method yields the highest Macro-precision, while the AVG method results in the lowest Macro-precision.

Additionally, the experiment compared the impact of different knowledge representation information *V*_*kg*_ and EMRs representation information *V*_*emrs*_ on information fusion methods. Under the same experimental conditions, we compared the average summation (AVG), weighted summation (WEIGHTED), and concatenation (CONCAT) fusion methods on the TCM-SD dataset. The experimental results, as shown in Figure 5, indicate that the CONCAT method achieved the best classification performance.

### 4.6 Compared with other methods

**Figure 6:**
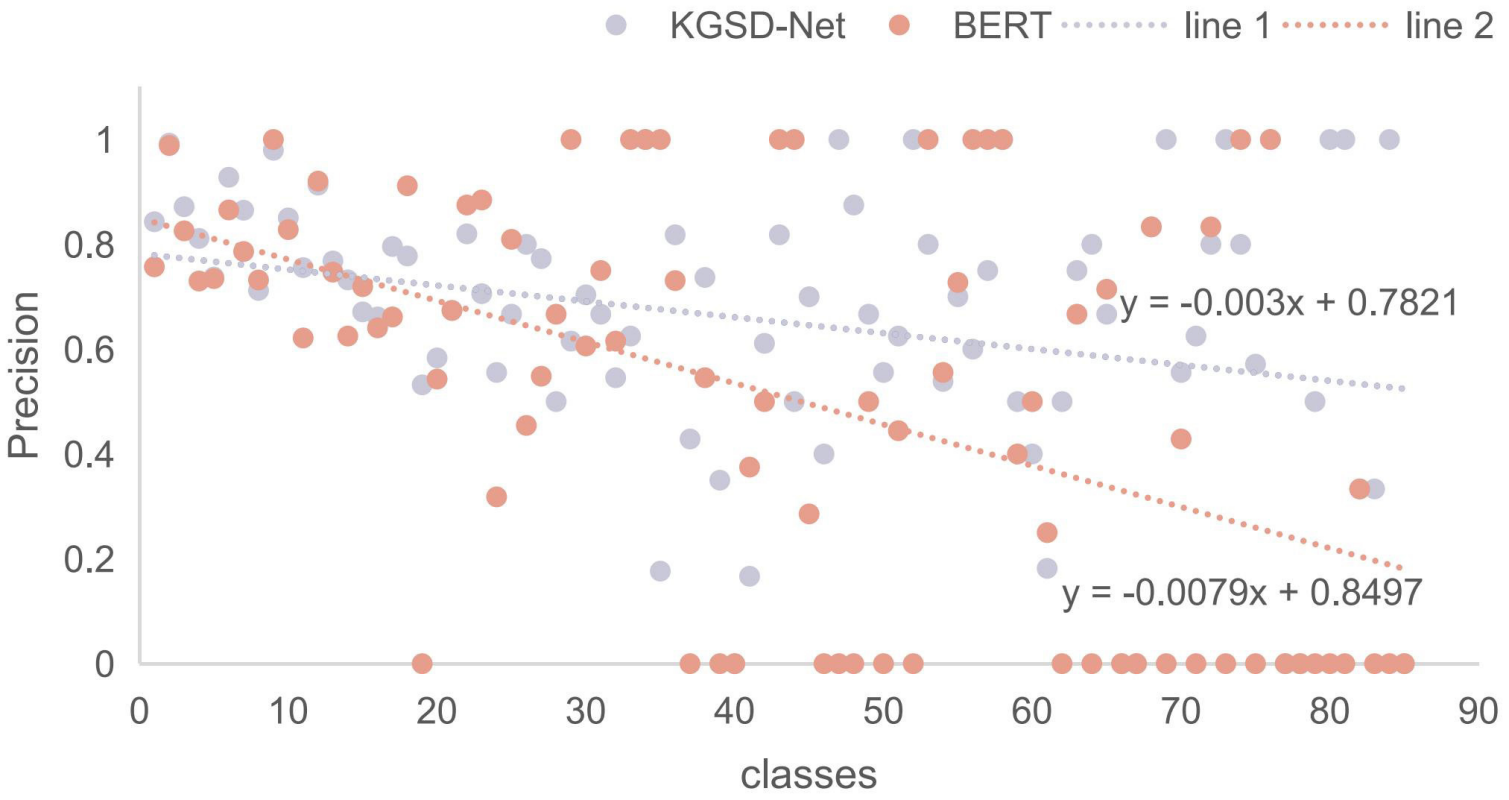
The precision for all categories is displayed, with the orange color representing the best result from BERT and the gray points indicating the precision of each category from our model in its best run. The line 1 and line 2 represent the fitted precision distribution functions for our model and BERT, respectively.

The baseline for experimental comparison includes three types of methods: those based on classical neural networks, methods leveraging language models, and models specifically designed for the TCM domain. The first category encompasses classical neural network models suitable for Chinese text categorization, including FastText (24), TextCNN (25), TextRNN (26), and Transformer. The second category focuses on classical models that have been effectively utilized for Chinese text categorization, such as BERT and Enrie (27). Afterwards, the third category features models tailored to the TCM domain; these utilize a domain-specific corpus for training to enhance performance on downstream tasks, such as TCM-sd. The last category is the multilingual general models that have performed well in various NLP tasks in recent years, such as E5 (28), and BGE-M3E (29). The results are shown in Table 6. The models showed less advantage here, even underperforming on precision compared to traditional deep learning models, likely due to limited exposure to TCM-specific corpora. Notably, the TCM-sd model, on a TCM corpus, outperformed in all metrics, highlighting the importance of domain-specific pre-training. Furthermore, we compare the classification precision of BERT and KGSD-Net across various syndrome classes. As illustrated in Figure 6, dataset imbalance results in variations in classification precision for certain classes. BERT faces challenges with classes that have fewer samples. In contrast, KGSD-Net enhances the classification of these low-sample classes, demonstrating its ability to deliver more stable classification outcomes across all classes compared to other models.

**Table 6:** Performance comparison of models on TCMSD dataset.

**Model**	**ACC**	**Macro-F1**	**Macro-precision**	**Macro-recall**
TextCNN (25)	0.7771	0.5000	0.6130	0.4734
TextRNN (26)	0.7604	0.3905	0.4346	0.3939
FastText (24)	0.7794	0.4788	0.6149	0.4490
Transformer (30)	0.7263	0.3852	0.5807	0.3443
BERT (31)	0.8014	0.5277	0.5576	0.5323
Enrie (27)	0.7814	0.4711	0.5405	0.4602
TCM-sd (32)	0.8104	0.5561	0.6352	0.5192
BGE-M3E (29)	0.8025	0.5430	0.5965	0.5251
E5 (28)	0.8200	0.5512	0.6218	0.5449
KGSD-Net	0.8259	0.5850	0.6518	0.5547

## 5 Conclusion

This paper presents advancements in TCM syndrome differentiation through the integration of LLMs and knowledge graph techniques. By extracting entities from unstructured data and constructing a dynamic TCM knowledge graph, a robust framework has been established that significantly enhances clinicians' capacity to make informed decisions in complex cases. The application of DeepWalk for latent embedding uncovers crucial patterns, while a combined entity linking approach effectively bridges traditional practices with electronic medical records, resulting in more coherent and structured diagnostic processes. Additionally, the incorporation of the BERT model enriches contextual understanding, ensuring that dialectical reasoning within TCM practice is both sophisticated and effective. Moving forward, future work will prioritize enriching the knowledge graph with diverse data sources to further refine accuracy in syndrome differentiation. Furthermore, optimization of the proposed network tailored specifically for clinical applications will be pursued alongside the exploration of personalized treatment recommendations based on individual patient profiles.

## Data Availability

The original contributions presented in the study are included in the article/supplementary material, further inquiries can be directed to the corresponding authors.
